# Supplementary material for: Establishment of Bactrian Camel Induced Pluripotent Stem Cells and Prediction of Their Unique Pluripotency Genes
Source: Int J Mol Sci. 2023 Jan 18;24(3):1917. doi: 10.3390/ijms24031917 (PMC9916525; doi:10.3390/ijms24031917)
Supplement: Supplementary file 1 [file ijms-24-01917-s001.zip › ijms-2135491-supplementary.pdf]

Supplementary Information

Establishment of Bactrian Camel Induced Pluripotent Stem Cells and Prediction of Their Unique Pluripotency Genes

Zongshuai Li[a,b], Yina Li[a,b], Qiran Zhang[b,c], Wenbo Ge[d],Yong Zhang[a, b,]*, Xingxu Zhao[a, b], Junjie Hu[a, b], Ligang Yuan[a] ,Wangdong Zhang[a]

[a]College of Veterinary Medicine, Gansu Agricultural University, Lanzhou 730070, china

[b]Gansu Key Laboratory of Animal Generational Physiology and Reproductive Regulation, Gansu Agricultural University, Lanzhou 730070, china

[c]College of Life Science and technology, Gansu Agricultural University, Lanzhou 730070, china90

[d] Chinese Academy of Agricultural Sciences Lanzhou Institute of Husbandry and Pharmaceutical Sciences, Lanzhou 730070, China

* Yong Zhang

Email:zhangyong@gsau.edu.cn

Table S1-S7

Table S1. Primers used in the construction of pMXS-EGFP and corrected pMXS-SOX2

Gene	Primer	Enzyme loci	Annealing temp(°C)	Fragment size(bp)
EGFP	TTT*AAGCTT*TTACTTGTACAGCTCGTCC<br>TAA*GGATCC*ATGGTGAGCAAGGGCGAG	HidⅢ<br>BamI	57	738
*EGF*P-J	GGTGGGGTCTTTCATTCC<br>CGCCGACACCAGACTAAG		57	1006
pMXC-F	CCG*CTCGAG*TTTAAATAGTACA	Xho Ⅰ	57	
Sox2-R1	TAA*GGATCC*CAGTGTGGTGGTACGGGA AATCACAAGTTTG	BamI	58	1174
Sox2-R2	CACAAGTTTGTACAAAAAAGCAGGCTA TGTATAACATGAT		58	1144
Sox2-jian	GGTGGGGTCTTTCATTCC<br>CGCCGACACCAGACTAAG		57	1430

Table S2. The composition of 6 different culture protocols

Protocol	OSKG	M	Trophoblast	VTN-N	E8	E8+LIF
1	√	√	√		√	
2	√	√	√			√
3	√	√		√	√	
4	√		√		√	
5	√		√			√
6	√			√	√	

O: Pseudovirus of *Oct4* gene

S: Pseudovirus of *Sox2* gene

K: Pseudovirus of *Klf4* gene

M: Pseudovirus of c-*Myc* gene

G: Pseudovirus of *EGFP* gene

Trophoblast: Mouse embryo fibroblasts treated with mitomycin C

VTN-N: Vitronectin (VTN-N) Recombinant Human Protein, Truncated

E8:Essential 8™ medium

LIF: 1000 U/ml leukemia inhibitory factor

Table S3. Primers used to detect endogenous genes and three germ layer genes

Gene	Primer	Annealing temperature(℃)	Fragment size(bp)
Endo *Sox2*	AGAGAACCCCAAGATGCACA<br>GCTTGGCCTCGTCGATAAAC	57	104
Endo *Nanog*	GATGCCTGGCGAATACTTCC<br>CTGCTGGGGACTGACATACT	63	329
FoxA2	ACAACCTCATGTCCTCGGAG<br>TAATGGGTCGGGAGTACACC	60	225
MYOD1	GGAGCTAGAGAGGACGAGCA<br>TCTCAAAGGCCTCGTTGACT	55	172
DES	CCATCGCGGCTAAGAACATC<br>GAATCGTTGGTGCCCTTGAG	55	184
MYF5	ACTTGCAAGGCTCAGACGAG<br>TGACCTTCTTCAGGCGTCTT	60	165
GFAP	ACATTGAGAAGGTGCGCTTC<br>TGTGCCAGATTGTCCCTCTC	55	190
PAX6	TCAGCACCAGTGTCTACCAG<br>AGGTATCATAACTCCGCCCG	55	243
TERT	AGAAGCTAGTCTCCCTGGGA<br>CCATCAACCAGCACAGGAAC	57	174
***β**-Actin*	CTGGACTTGGAGCAGGAGATG<br>CACCGTGTTGGCGTAGAGG	55	234

Table S4. Primers used to detect RNA-Seq sequencing data

Gene	Primer	Annealing temperature(℃)	Fragment size(bp)
CCNB2	AGTACCTGAGGCAGCTTGAG<br>GCAGAGCTGTAATCCCAACC	53	231
CDC20	AGAATCAGCCCGAAAACAGC<br>AGGGAAGGAATGTAACGGCA	57	220
ICAM1	TGATACCCTCCGGATTGTGG<br>ATTCTCCAGTCTACTCCGCG	56	247
IFK67	CCGTCATACCCAGCAGTGAA<br>CCTGGCGCTTTCTGGAATTT	56	208
MMP9	GCAAACCAATCTCACCGACA<br>GGTCAGAAATTTGCCCACGT	53	223
PCNA	CGGACACCTTGGCACTAGTA<br>CACCCCATCTTTTGCACAGG	56	215
PLK1	CTCGACACGCCTTATCCTCT<br>GGGCTAGCTCATCACCTTCA	53	206
RRM2	TGCCTGCCTGATGTTCAAAC<br>GCATAAGTCTGTCTGCCACG	53	182
S100A4	TTCCACAAGTACTCGGGCAA<br>TTATCCGGGAAGCCTTCGAA	56	242
TP3	GAGGACGCCAGTGGTAATCT<br>AGCGGCTTCTTCTTTTGTGG	56	197
VEGFC	CTCTCTCTCAAGGCCCCAAA<br>GGGTCATCTCCAGCGTTAGA	53	244
VIM	GATTCAGGAACAGCACGTCC<br>TTGGATTCTTGCTTCGCCTG	56	207

Table S5. Inducible genes collection

Gene	Author	Gene	Author	Gene	Author
c-Myc	Takahashi K et al[1]	*Sox15*	Nakagawa M et al[2]	*PAX1*	Shu J et al [3]
L-Myc	Nakagawa M et al[4]	*Sox18*	Nakagawa M et al[2]	*GATA4*	Shu J et al[3]
N-Myc	Nakagawa M et al[4]	*Rcor2*	Yang P et al[5]	*CEBPa*	Shu J et al[3]
Glis1	Maekawa M et al[6]	*Obox1*	Wu L et al[7]	*HNF4a*	Shu J et al[3]
Klf4	Takahashi K et al[1]	*GMNN*	Shu J et al[3]	*GRB2*	Shu J et al[3]
Klf1	Lee B C et al[8]	*TH2A*	Toshie et al[9]	*Tet1*	Gao Y et al[10]
Klf2	Lee B C et al[8]	*TH2B*	Toshie et al[9]	*Jhdm1b*	Liu J et al[11]
Klf5	Lee B C et al[8]	*Oct4*	Takahashi K et al[1]	*Bmi1*	Moon JH et al[12]
Esrrb	Bo F et al[13]	*Nr5a1*	Heng J et al[14]	*Sall4*	Liu J et al[11]
Bmp4	Chen J et al[15]	*Nr5a2*	Heng J et al[14]	*Dppa2*	Buganim Y et al[16]
Nanog	Yu J et al[17]	*CDH1*	Redmer T et al[18]	*Lrh1*	Liu J et al[11]
Lin28	Yu J et al[17]	*TCL-1A*	Picano-Castro V et al[19]	*Jdp2*	Liu J et al[11]
Sox2	Takahashi K et al[1]	*Brn4*	Bar-Nur O et al[20]	*Id1*	Liu J et al[11]
Sox1	Nakagawa M et al[2]	*GATA6*	Shu J et al[3]		
Sox3	Nakagawa M et al[2]	*Sox7*	Shu J et al[3]		

Table S6. Annotation information in the GO term and KEGG pathway where the induced gene is located

Database	Detailed information
Pathway	ko04550//Signaling pathways regulating pluripotency of stem cells<br>ko04390//Hippo signaling pathway<br>ko05200//Pathways in cancer<br>ko04350//TGF-beta signaling pathway<br>ko04218//Cellular senescence<br>ko05202//Transcriptional misregulation in cancers<br>ko04950//Maturity onset diabetes of the young<br>ko04934//Cushing syndrome
GO Component	GO:0043231//intracellular membrane-bounded organelle<br>GO:0043232//intracellular non-membrane-bounded organelle<br>GO:0044451//nucleoplasm part<br>GO:0090575//RNA polymerase II transcription factor complex<br>GO:0044424//intracellular part
GO Function	GO:0000982//transcription factor activity, RNA polymerase II core promoter proximal region sequence-specific binding<br>GO:0000977//RNA polymerase II regulatory region sequence-specific DNA binding<br>GO:0003712//transcription cofactor activity<br>GO:0046983//protein dimerization activity<br>GO:0003723//RNA binding<br>GO:0000976//transcription regulatory region sequence-specific DNA binding<br>GO:0008134//transcription factor binding<br>GO:0005515//protein binding<br>GO:0046914//transition metal ion binding<br>GO:0001071//nucleic acid binding transcription factor activity<br>GO:0043169//cation binding<br>GO:0000975//regulatory region DNA binding<br>GO:0003700//transcription factor activity, sequence-specific DNA binding<br>GO:0000981//RNA polymerase II transcription factor activity, sequence-specific DNA binding<br>GO:0003677//DNA binding<br>GO:0003676//nucleic acid binding<br>GO:0019904//protein domain specific binding
GO Process	GO:0001101//response to acid chemical<br>GO:0006357//regulation of transcription from RNA polymerase II promoter<br>GO:0001708//cell fate specification<br>GO:0019827//stem cell population maintenance<br>GO:0008284//positive regulation of cell proliferation<br>GO:0003006//developmental process involved in reproduction<br>GO:0045597//positive regulation of cell differentiation<br>GO:0060795//cell fate commitment involved in formation of primary germ layer<br>GO:0008285//negative regulation of cell proliferation<br>GO:0008283//cell proliferation<br>GO:0042981//regulation of apoptotic process<br>GO:0006355//regulation of transcription, DNA-templated<br>GO:0043009//chordate embryonic development<br>GO:0010468//regulation of gene expression
No information	No information

References

[1]Takahashi K, Yamanaka S. Induction of pluripotent stem cells from mouse embryonic and adult fibroblast cultures by defined factors [J]. Cell, 2006, 51(4): 2346-2351.

[2]Nakagawa M, Koyanagi M, Tanabe K, et al. Generation of induced pluripotent stem cells without Myc from mouse and human fibroblasts [J]. Nature Biotechnology, 2008,

[3]Shu J, Wu C, Wu Y, et al. Induction of Pluripotency in Mouse Somatic Cells with Lineage Specifiers [J]. Cell, 2015, 153(5): 963-975.

[4]Nakagawa M, Takizawa N, Narita M, et al. Promotion of direct reprogramming by transformation-deficient Myc [J]. Proceedings of the National Academy of Sciences of the United States of America, 2010, 107(32): 14152-14157.

[5]Yang P, Wang Y, Chen J, et al. RCOR2 Is a Subunit of the LSD1 Complex That Regulates ESC Property and Substitutes for SOX2 in Reprogramming Somatic Cells to Pluripotency [J]. Stem Cells, 2011, 29(5):

[6]Maekawa M, Yamaguchi K, Nakamura T, et al. Direct reprogramming of somatic cells is promoted by maternal transcription factor Glis1 [J]. Nature, 2011 Jun 8, 474(7350): 225-229.

[7]Wu L, Wu Y, Peng B, et al. Oocyte-Specific Homeobox 1, Obox1 , Facilitates Reprogramming by Promoting Mesenchymal-to-Epithelial Transition and Mitigating Cell Hyperproliferation [J]. Stem Cell Reports, 2017, 9(5):

[8]Lee B C, Kim M K, Jang G, et al. Dogs cloned from adult somatic cells [J]. Nature, 2005, 436(7051): 641.

[9]Toshie, Shinagawa, Tsuyoshi, et al. Histone Variants Enriched in Oocytes Enhance Reprogramming to Induced Pluripotent Stem Cells [J]. Cell Stem Cell, 2014, 14(2): 217-227.

[10]Gao Y, Chen J, Li K, et al. Replacement of Oct4 by Tet1 during iPSC Induction Reveals an Important Role of DNA Methylation and Hydroxymethylation in Reprogramming [J]. Cell Stem Cell, 2013, 12(4): 453-469.

[11]Liu J H Q, Peng T, Peng M, Wei B, Li D. The oncogene c-Jun impedes somatic cell reprogramming [J]. Nat Cell Biol, 2015, 17(7): 856-867.

[12]Moon JH H J, Kim JS, Jun EK, Lee JH, Kim A, et al. Reprogramming fibroblasts into induced pluripotent stem cells with Bmi1 [J]. Cell Research, 2011, 21(9): 1305-1315.

[13]Bo F, Jiang J, Kraus P, et al. Reprogramming of fibroblasts into induced pluripotent stem cells with orphan nuclear receptor Esrrb [J]. Nature Cell Biology, 2009, 11(2): 197-203.

[14]Heng J, Feng B, Han J, et al. The nuclear receptor Nr5a2 can replace Oct4 in the reprogramming of murine somatic cells to pluripotent cells [J]. Cell Stem Cell, 2010, 6(2): 167-174.

[15]Chen J, Liu J, Yang J, et al. BMPs functionally replace Klf4 and support efficient reprogramming of mouse fibroblasts by Oct4 alone [J]. Cell Research, 2011, 21(1): 205-212.
[16]Buganim Y, Faddah D, Cheng A, et al. Single-Cell Expression Analyses during Cellular Reprogramming Reveal an Early Stochastic and a Late Hierarchic Phase [J]. Cell, 2012, 150(6): 1209-1222.
[17]Yu J, Vodyanik M A, Smuga-Otto K, et al. Induced Pluripotent Stem Cell Lines Derived from Human Somatic Cells [J]. Science, 2007, 318(5858): 1917–1920.
[18]Redmer T, Diecke S, Grigoryan T, et al. E-cadherin is crucial for embryonic stem cell pluripotency and can replace OCT4 during somatic cell reprogramming [J]. Embo Reports, 2011, 12(7): 720-726.
[19]Picano-Castro V, Russo-Carbolante E, Reis L, et al. Pluripotent reprogramming of fibroblasts by lentiviral mediated insertion of SOX2, C-MYC, and TCL-1A [J]. Stem Cells & Development, 2011, 20(1): 169-180.
[20]Bar-Nur O, Verheul C, Sommer A G, et al. Lineage conversion induced by pluripotency factors involves transient passage through an iPSC stage [J]. Nature Biotechnology, 2015, 33(7): 761-768.
